# Cationic Porphyrins as Effective Agents in Photodynamic Inactivation of Opportunistic Plumbing Pathogen *Legionella pneumophila*

**DOI:** 10.3390/ijms21155367

**Published:** 2020-07-28

**Authors:** Andrija Lesar, Martina Mušković, Gabrijela Begić, Martin Lončarić, Dijana Tomić Linšak, Nela Malatesti, Ivana Gobin

**Affiliations:** 1 Bioinstitut d.o.o., R. Steinera 7, 40000 Čakovec, Croatia; lesar@bioinstitut.hr; 2 Department of Biotechnology, University of Rijeka, Radmile Matejčić 2, 51000 Rijeka, Croatia; martina.muskovic@biotech.uniri.hr; 3 Department of Microbiology and Parasitology, Faculty of Medicine, University of Rijeka, Braće Branchetta 20, 51000 Rijeka, Croatia; gabrijela.begic@uniri.hr (G.B.); ivana.gobin@uniri.hr (I.G.); 4 Photonics and Quantum Optics Unit, Center of Excellence for Advanced Materials and Sensing Devices, Ruđer Bošković Institute, Bijenička cesta 54, 10002 Zagreb, Croatia; martin.loncaric@irb.hr; 5 Department for Health Ecology, Faculty of Medicine, University of Rijeka, Braće Branchetta 20, 51000 Rijeka, Croatia; dijanatl@medri.uniri.hr

**Keywords:** photodynamic inactivation, porphyrins, photosensitisers, antibacterial activity, *Legionella pneumophila*, *Acanthamoeba castellanii*

## Abstract

*Legionella pneumophila* is an environmental bacterium, an opportunistic premise plumbing pathogen that causes the Legionnaires’ disease. *L. pneumophila* presents a serious health hazard in building water systems, due to its high resistance to standard water disinfection methods. Our aim was to study the use of photodynamic inactivation (PDI) against *Legionella*. We investigated and compared the photobactericidal potential of five cationic dyes. We tested toluidine blue (TBO) and methylene blue (MB), and three 3-*N*-methylpyridylporphyrins, one tetra-cationic and two tri-cationic, one with a short (CH_3_) and the other with a long (C_17_H_35_) alkyl chain, against *L. pneumophila* in tap water and after irradiation with violet light. All tested dyes demonstrated a certain dark toxicity against *L. pneumophila*; porphyrins with lower minimal effective concentration (MEC) values than TBO and MB. Nanomolar MEC values, significantly lower than with TBO and MB, were obtained with all three porphyrins in PDI experiments, with amphiphilic porphyrin demonstrating the highest PDI activity. All tested dyes showed increasing PDI with longer irradiation (0–108 J/cm^2^), especially the two hydrophilic porphyrins. All three porphyrins caused significant changes in cell membrane permeability after irradiation and *L. pneumophila*, co-cultivated with *Acanthamoeba castellanii* after treatment with all three porphyrins and irradiation, did not recover in amoeba. We believe our results indicate the considerable potential of cationic porphyrins as effective anti-*Legionella* agents.

## 1. Introduction

Opportunistic plumbing pathogens (OPPPs) constitute a group of microbial residents in premise plumbing water distribution systems (water pipes in houses, hotels, hospitals etc.). They share characteristics such as biofilm formation, resistance to disinfectants, growth at low organic and oxygen levels and survival in amoebae [1]. The OPPP group includes numerous pathogen bacteria such as *Legionella pneumophila*, *Pseudomonas aeruginosa*, *Mycobacterium avium* complex, *Methylobacterium* spp., *Acinetobacter baumanii*, and *Stenotrophomonas* spp. [2].

Standard methods for water disinfection include physical and chemical treatments such as chlorination or oxidation, use of biocides and treatments of a physical nature (UV irradiation and thermal disinfection). *Legionella*, and other OPPPs, represent serious a public health threat as they are highly resistant to standard water disinfection methods in comparison to other microorganisms [3]. For instance, it was shown that *Legionella* was still present in water systems after repeated use of standard treatment of chlorination (50 g/L) [4] or/and thermal disinfection (60–65 °C) [5].

To overcome problems associated with resistant strains and inefficient methods of disinfection, photodynamic inactivation (PDI) has been increasingly applied as a new tool for microbial destruction [6]. It acts as a multi-targeted process, where, upon light irradiation, a photosensitizer (PS) gives energy for conversion of molecular oxygen into a cytotoxic singlet state of oxygen (^1^O_2_) [7]. Singlet oxygen can cause bacterial cell death through several mechanisms that involve oxidative damage to nucleic acids, amino acids in proteins and membrane lipids [6].

Among photosensitizers with a tetrapyrrole macrocycle core, such as chlorins, bacteriochlorins, phthalocyanines and porphyrins, which may all be utilised for a photodynamic therapy (PDT), porphyrins are so far the most used and investigated PSs [6]. Cationic porphyrins are an especially investigated group of PSs, because they show similar high effectiveness against Gram positive bacteria as neutral or anionic porphyrins, but are advantageous over them in the treatments against Gram negative bacteria strains. In fact, they interact electrostatically with lipopolysaccharides (LPS) in their membrane, which in turn results in an increased binding affinity for bacterial cells [8].

Porphyrins, as PDI agents, have been tested against various OPPPs, such as the life-threatening bacteria *Pseudomonas aeruginosa* [9,10], and *Mycobacteria* [11]. *Enterococcus faecalis* and *Escherichia coli* [12,13], the two pathogens present in environmental water, have also been highly investigated with PDI. Furthermore, there are examples of silica-gel and other micro- or nanoparticle immobilized porphyrins being used for photosensitized disinfection of water [14], such as 5,10,15,20-tetraphenylporphyrin (TPP) and cationic *meso*-tetra(*N*-methylpyrid-4-yl)porphyrin (TMPyP) encapsulated in polysilsesquioxanes, which is used against *E. coli* in water [15]. However, to the best of our knowledge, there are only few examples of photodynamic actions against *Legionella pneumophila*, a Gram-negative non-fermenting bacterium that causes a severe type of pneumonia, Legionnaire’s disease, such as [16], a study that suggested a photobactericidal approach to water treatments against *Legionella*. In one of them, the high bactericidal activity of silica gel-supported metalloporphyrin against *Legionella* has been demonstrated, but only with practical experiments in a water fountain [17]. This motivated us to initiate our in vitro studies of PDI using cationic amphiphilic porphyrin as a PS against *L. pneumophila* [18].

Our aim here was to study further, look into more details, and compare the PDI activity of three cationic porphyrins against *L. pneumophila*. Widely used cationic phenothiazinium photosensitizers, toluidine blue O (TBO) and methylene blue (MB) were also applied in PDI experiments in water as a comparison of their antibacterial activities with those of cationic porphyrins. A total of two known hydrophilic cationic porphyrins bearing three and four positive charges were prepared for this study, as well as a previously studied amphiphilic porphyrin with a long alkyl chain [18,19].

## 2. Results and Discussion

### 2.1. Synthesis of Porphyrins

**Scheme 1:**
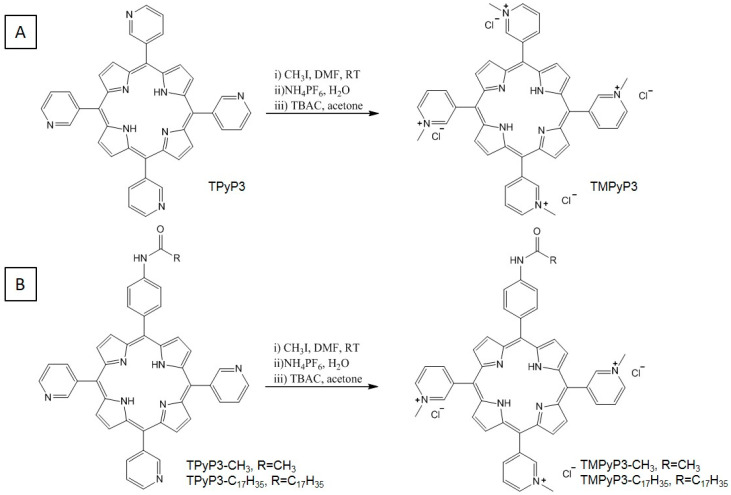
*N*-methylation of (**A**) tetra- and (**B**) tripyrid-3-ylporphyrins using a NH_4_PF_6_/tetrabuthylammonium chloride (TBAC) precipitation method.

The synthesis of a series of *N*-methylated tri- and tetrapyrid-3-ylporphyrins was previously described by our group [19,20]. However, to improve ion exchange and purification after the methylation reaction using methyl iodide, we applied a modified methylation method using ammonium hexafluorophosphate (NH_4_PF_6_)/tetrabuthylammonium chloride (TBAC) precipitations, according to procedures described in the literature [21]. Previously we used Amberlite IRA 400 for an ion exchange (I^−^ to Cl^−^) after methylation with CH_3_I. However, those reactions had difficulties in reaching completion, and the reaction mixtures were often very difficult to purify. A precipitation method using NH_4_PF_6_/TBAC led to a complete ion exchange, with faster and easier purification of TMPyP3, TMPyP3-CH_3_ and TMPyP3-C_17_H_35_ (Scheme 1).

### 2.2. Photophysical Properties of Synthetized Porphyrins

**Figure 1:**
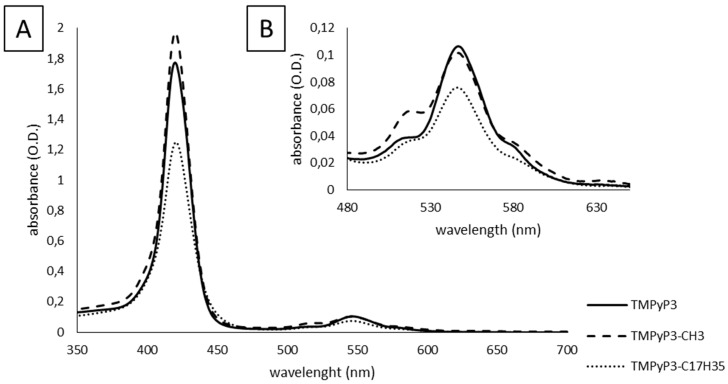
Comparison of (**A**) UV–Vis absorption spectra and (**B**) Q-bands of UV–Vis absorption spectra (enlarged), of porphyrins TMPyP3, TMPyP3-CH_3_ and TMPyP3-C_17_H_35_ (10 µM) in tap water.

**Figure 2:**
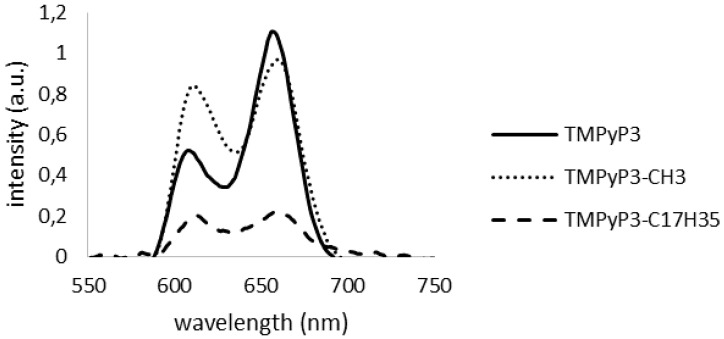
Comparison of fluorescence spectra of TMPyP3, TMPyP3-CH_3_ and TMPyP3-C_17_H_35_ (2 µM) obtained by excitation at Soret band wavelength. All measurements were performed on tap water of the city of Rijeka.

All the obtained *N*-methylated porphyrins are soluble in water. Previously, we showed their absorption and fluorescence spectra recorded in methanol [19]; here, we show the absorption (Figure 1) and fluorescence (Figure 2) spectra obtained in tap water used for studies of their activity against *Legionella*. In comparison to spectra collected in methanol and distilled water (Figures S4–S6), the absorption spectra recorded in tap water do not show clearly all four Q bands characteristic for free base porphyrins. Rather, they resemble the spectra of metalloporphyrins. This resemblance may be explained by the presence of various cations in tap water (Table 1) and their interactions.

Furthermore, slightly lower molar absorption coefficients at Soret band were obtained for the porphyrins dissolved in tap water (Table 2), in comparison to their values obtained in methanol [19]. A difference can be seen also between TMPyP3-CH_3_ and TMPyP3-C_17_H_35_, with a TMPyP3-C_17_H_35_ spectrum that shows a higher molar absorption coefficient at Soret band than TMPyP3-CH_3_ when recorded in methanol, while in tap water the molar absorption coefficient of TMPyP3-C_17_H_35_ (180.0 × 10^3^ M^−1^cm^−1^) was lower than the molar absorption coefficient of TMPyP3-CH_3_ (204.5 × 10^3^ M^−1^·cm^−1^). This can be explained by their respective solubility; TMPyP3-C_17_H_35_ is more lipophilic, thus more soluble in methanol, while TMPyP3-CH_3_ is more hydrophilic, thus more soluble in water.

Differences in fluorescence spectra can also be observed, for example fluorescence of TMPyP3-C_17_H_35_ could not be detected at 1 µM concentration, and generally, the intensity of fluorescence for all the porphyrins was lower in water than in methanol. The presence of metal cations in tap water may also have an effect on the porphyrins’ fluorescence spectra, observed as a hypsochromic shift in tap water, such as for TMPyP3-CH_3_: λ_em_(in H_2_O_tap_)/nm = 613, 660 and TMPyP3-C_17_H_35_: λ_em_(in H_2_O_tap_)/nm = 612, 660 (Figure 2), compared to their emission peaks in methanol, for both λ_em_(in MeOH)/nm = 651, 714 [19].

### 2.3. Physicochemical Properties of the Used Tap Water

Since the interaction of porphyrins and different minerals may occur and influence their PDI activity, the physical and chemical parameters of the tap water have been characterized. Physicochemical parameters of water for human consumption in Rijeka district are regularly monitored and show values that rarely deviate. Water is colourless and odourless with a normal temperature parameter which follows seasonal variations. It has low turbidity, neutral to slightly alkaline pH, low conductivity values, low evaporation residues and total suspension; it has moderate total hardness.

According to the degree of mineralization, the water is of moderate hardness. Hydrogen carbonates of calcium and magnesium are predominant in water, with low concentrations of sodium, potassium, chloride, fluoride, sulphate, and other measured ions (Table 2).

### 2.4. Minimal Effective Concentration (MEC) and Porphyrin Uptake

Toluidine blue O (TBO) and methylene blue (MB) are phenothiazine dyes that are extensively used as PSs in PDI due to the low toxicity and positive charge in their structures, that renders them effective against Gram positive as well as negative bacteria [22,23]. In a study by Usacheva et al., both dyes, MB and TBO, demonstrated bactericidal activity in dark as well as under light conditions against tested Gram positive and Gram negative pathogenic bacteria (the study did not include *Legionella*) [24]. However, TBO was more efficient than MB against different bacteria in both conditions, with and without light. Both dyes are hydrophilic, with partition coefficients 0.33 and 0.11 for TBO and MB, respectively, and longer incubation increased their photobactericidal efficacy against tested Gram negative. The authors suggested that, although both dyes efficiently bind to the outside membrane, they cause the damage to it upon irradiation; TBO is more efficient due to higher hydrophobicity and the ability to penetrate into the plasma membrane [24].

Some PDI applications in water disinfection could potentially use solar energy. Nevertheless, violet and blue light penetrate the deepest into water. Thus, we decided to use violet light of known fluence for our anti-*Legionella* studies on local tap water. We proceeded in this way also to be able to calculate the light doses corresponding to the time of irradiation. In this spectrum, TBO and MB have a very small light absorption, as their respective *λ*_max_ values are 631 nm and 665 nm [24], while all tested porphyrins have strong absorption with their Soret band around 420 nm [20]. The obtained MEC values (Table 3) indicate a somewhat dark toxicity for all tested dyes after incubation with *L. pneumophila* for 30 min, the highest being observed for the compound with a long alkyl chain, TMPyP3-C_17_H_35_ (>1.56 µM), while the other two porphyrins that are hydrophilic show two-fold dark toxicity (>12.5 µM) in comparison to hydrophilic phenothiazine dyes TBO and MB (>25.6 µM). The mechanism of self-promoted uptake, which is due to interactions between the positive charges of cationic PSs and negative charges of LPS, has been used previously to explain similar dark toxicity [25]. Even though the experiment was performed in dark, the determination of the colony forming units (CFU) could not be carried out in complete darkness. This might explain the observed significant “dark” toxicity for TMPyP3-C_17_H_35_. This was also the compound with the highest photoactivity among those that were tested, as we describe in the following paragraph.

After 30 min incubation of *L. pneumophila* with PSs and a subsequent 10 min of irradiation, the obtained MEC values were lower for all tested dyes in comparison to their dark toxicities. A two-fold higher photobactericidal activity was achieved with TBO compared to MB, which is in accordance with the aforementioned observations [24]. All the porphyrins had significantly lower MEC values than TBO and MB, and again, the highest photobactericidal efficacy was achieved with TMPyP3-C_17_H_35_ (0.024 µM) (also measured previously in [18]), that is ~1000 times more efficient than MB, which is a dye with the highest MEC value. Amongst hydrophilic porphyrins, tetra-cationic TMPyP3 was more PDI efficient than tri-cationic TMPyP3-CH_3_ (Table 3).

The impact of positive charges, their number and distribution in a molecule, has been previously investigated, mostly on *E. coli* and *S. aureus* [8,12,26,27]. Recently, a tetra-cationic porphyrin similar to TMPyP3 was reported to have higher PDI activity compared to the tri-cationic porphyrin, and was shown to be internalized by *E. coli*, while the tri-cationic bound to the cellular membrane [25]. On the other hand, tetra-cationic porphyrins are also known to interact strongly with nucleic acids, causing photodamage to plasmid DNA for instance, while tri-cationic porphyrins target lipids in the cell membrane [25]. This could also explain the strongest PDI activity of significantly more lipophilic tri-cationic porphyrin TMPyP3-C_17_H_35_, as opposed to hydrophilic tri-cationic porphyrin TMPyP3-CH_3_. We also observed the impact of a long alkyl chain in TMPyP3-C_17_H_35_ and high photodynamic activity in comparison to a hydrophilic porphyrin against various tumour cell lines [19,21], and other authors reported a similar effect in PDI against various Gram positive and Gram negative bacteria strains [8,12,28]. It was suggested that the alkyl chain can help as a hydrophobic arm inserted in the external part of the bacterial membrane to stabilize the porphyrin on the membrane and additionally weaken it [9]. *L. pneumophila* contains a complex and unusual LPS structure different from other Gram negative bacteria [29], and this might explain the high PDI activity of TMPyP3-C_17_H_35_ against *L. pneumophila* in comparison to the low activity obtained with the same PS against other Gram negative bacteria (unpublished results).

The main bacterial targets of PDI are the outer cell structure and membrane lipids [30,31]. As previously mentioned, the binding of cationic PSs to cells of Gram negative bacteria is much stronger than those of neutral and anionic PSs, and with porphyrins the binding is higher for PSs with a higher number of positive charges [28,32]. Furthermore, it has been shown that the amphiphilic structure and the highly lipophilic group in the tri-cationic porphyrin structure significantly increase binding affinity [32], and a longer chain may also facilitate penetration of the PS into the cytosol of the cells [28,33].

Furthermore, we tested the incubation time required for the bacterial cell uptake, and as it was previously shown for TMPyP3-C_17_H_35_ [18]; TMPyP3 and TMPyP3-CH_3_ have also bound to *Legionella* right after 10 min of incubation (Figure S7). No statistical difference was observed with prolonged incubation of all porphyrins with bacteria.

It was previously found that cationic porphyrins can bind to the bacterial cell wall within 5–10 min, and a prolonged incubation time did not show any significant difference in the number of bacteria [13,32,34]. Moreover, tri- and tetra-cationic porphyrins proved to be bound firmly to *E. coli*, even after several washing steps [34].

### 2.5. Photodynamic inactivation of Legionella pneumophila

**Figure 3:**
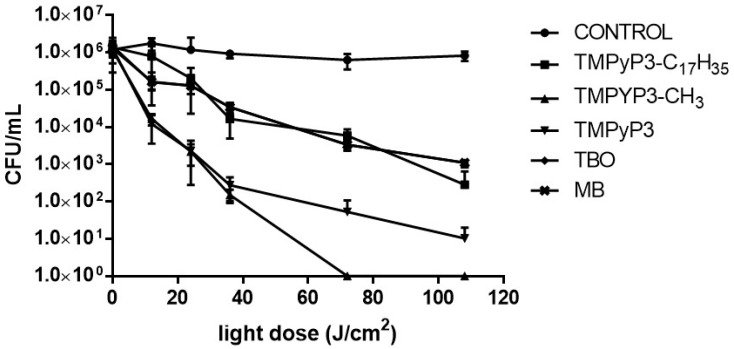
Photodynamic inactivation of *L. pneumophila* (CFU/mL) in the presence of 0.5× MEC of photosensitizers (PSs) exposed to different doses of violet light (*λ* = 394 nm; irradiance 20 mW·cm^−2^). The control is *L. pneumophila* that was exposed to different light doses, without treatment with PSs. Data are given as the mean of 3 independent experiments ± standard deviation (SD).

For the analysis of *L. pneumophila* cultivability after treatment with PSs, and to study the effect of light doses (12, 24, 36, 72 and 108 J/cm^2^) through increasing irradiation times of (10, 20, 30, 60 and 90 min, respectively), we examined the MEC values of each porphyrin, although their concentrations differ. Thus, we examined the effect of bactericidal doses of porphyrins, the cell killing rate of every porphyrin and whether the bactericidal effect is drug/light dose dependent. PDI experiments were performed, with 0.5, 1 and 2 MEC values of each porphyrin (Figure 3, Figures S8 and S9). To serve as a control, *Legionella* was exposed only to light, without adding PSs. Light at any dose, i.e., irradiation time, did not affect bacterial cultivability.

All tested porphyrins exhibited a significant decrease of CFU/mL value that was light dose dependent, thus a strong photobactericidal activity against *L. pneumophila* can be observed already at low doses of violet light and low concentrations (0.5× MEC) in comparison to controls (CFU/mL (at 108 J/cm^2^ final dose of light) = 7.95 × 10^5^ ± 1.39 (Figure 3).

The tri-cationic hydrophilic porphyrin TMPyP3-CH_3_ inactivated 100% of *L. pneumophila* already at 0.5× MEC (0.195 µM) concentration at light dose ~70 J/cm^2^. A high PDI activity was observed also for tetra-cationic TMPyP3, where a ~5 log decrease in cell cultivability was detected at 108 J/cm^2^ light dose and conc. of 0.0975 µM. Increase in the PDI activity dependent on light dose was very similar between TMPyP3-CH_3_ and TMPyP3 up to doses of 36 J/cm^2^, but became higher for the tri-cationic porphyrin with higher light doses. Considering that the MEC value was somewhat higher for the tri-cationic porphyrin TMPyP3-CH_3_ (Table 3), and taking into account previous observations that similar tri-cationic porphyrins bound to the outer cell structures while tetra-cationic internalized, this could indicate that with a higher light dose the extent of the cell membrane damage makes it more permeable for TMPyP3-CH_3_, increasing the overall impact of PDI [31]. The results obtained for TMPyP3-C_17_H_35_ are in the agreement with our previously described PDI studies with the same compound [18]. In this study, amphiphilic porphyrin TMPyP3-C_17_H_35_ also showed high PDI activity (~3 log decrease at 108 J/cm^2^), and PDI dependence on the light dose, at a 0.5× MEC concentration value compared to those of both MB and TBO; however, it has to be pointed out that MEC values for MB and TBO were significantly higher (Table 3).

At higher concentrations (1× MEC and 2× MEC), of all three tested porphyrins, TMPyP3 (for both concentrations at 36 J/cm^2^), TMPyP3-CH_3_ (at 36 J/cm^2^ and 24 J/cm^2^), and TMPyP3-C_17_H_35_ (at 108 J/cm^2^ and 72 J/cm^2^) exhibited complete *L. pneumophila* inactivation. TBO reached complete inactivation only at maximal tested values, 2× MEC and 108 J/cm^2^, while MB remained the least PDI active compound among all those tested (Figures S8 and S9).

To summarize the previous results, from the collected data, the light dose required for 50% inhibition of *Legionella* cells was calculated (Table 4). For all tested porphyrins, increasing concentration two-fold from 0.5× MEC to 1× MEC value, reduced ~three-fold the required light dose for 50% inhibition, and increasing two-fold from 1× MEC to 2× MEC reduced it ~1.1 times. All the values in Table 4 that were obtained for amphiphilic TMPyP3-C_17_H_35_ were 3–3.5 times higher than those for two hydrophilic porphyrins, but it has to be pointed out that the MEC value for TMPyP3 was ~8, and for TMPyP3-CH_3_ even 16 times higher, than for TMPyP3-C_17_H_35_. Finally, both high concentrations of PS and high light doses were necessary for 50% inhibition of *Legionella* with MB. However, TBO was more effective than MB, especially at 1× and 2× MEC concentrations when the required light dose for 50% inhibition was reduced from 104.73 (with 0.5× MEC) to 62.25 and 26.72 J/cm^2^, respectively.

Since phenothiazine dyes TBO and MB showed only moderate and low PDI activity against *L. pneumophila*, we decided not to use them in further PDI experiments.

### 2.6. Viability of Legionella pneumophila after Treatment with PSs

The permeability of the bacterial cell membrane was tested with SYTO-9 and propidium iodide (PI) staining. SYTO-9 is a stain used for green fluorescence, and appears when the cell membrane is intact, denoting viable cells. On the other hand, red fluorescence is shown when bacterial cells have damaged membranes and are stained with PI.

**Figure 4:**
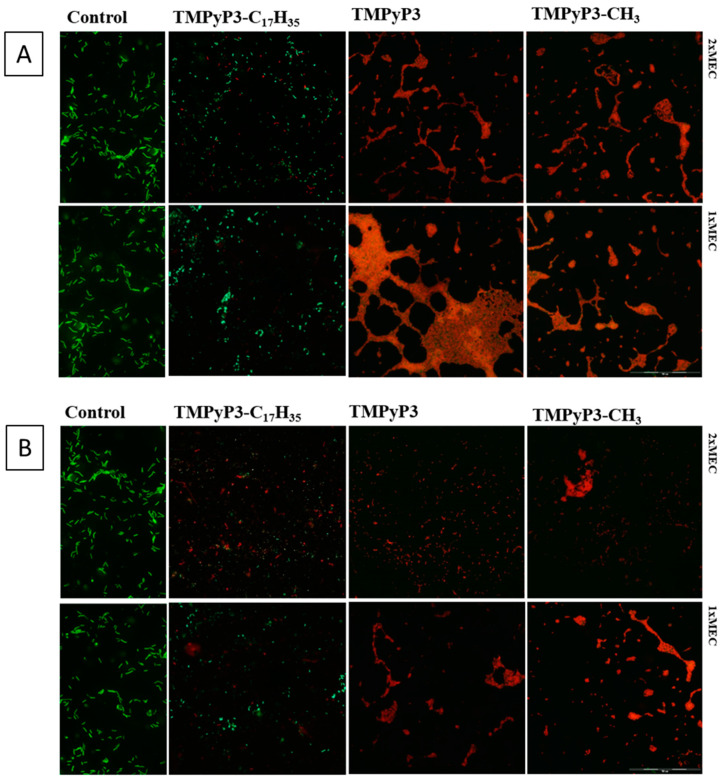
Changes in membrane permeability, observed under fluorescent microscope. *Legionella pneumophila* was treated with PSs of 1× MEC and 2× MEC concentrations and after 30 min incubation protected from light, illuminated with violet light for 10 min (**A**) and 30 min (**B**) (total light dose 12 J/cm^2^ and 36 J/cm^2^, respectively). Membrane permeability was tested using Dead Live staining. Green fluorescence is representing an intact membrane, and red fluorescence indicates a permeable membrane. Scale bars correspond to 50 μm.

Our results showed that after irradiation for 10 min using porphyrins TMPyP3 and TMPyP3-CH_3_, in both concentrations the major part of the membranes was damaged, shown by red fluorescence (Figure 4A). However, after using TMPyP3-C_17_H_35_ in both concentrations and light exposure for 10 min, a minor number of bacterial cells was shown by red fluorescence, indicating a large number of viable cells.

After a 30 min treatment with violet light (Figure 4B), a higher number of bacteria with red fluorescence was observed for TMPyP3-C_17_H_35_, indicating a higher number of dead cells in both concentrations. Furthermore, an increased number of bacteria with red fluorescence was also observed for porphyrins TMPyP3 and TMPyP3-CH_3_ in 1× MEC concentration. However, in a 2× MEC concentration, no bacteria with green fluorescence were observed, indicating there were no viable cells after 30 min (light dose 36 J/cm^2^) exposure to violet light.

**Figure 5:**
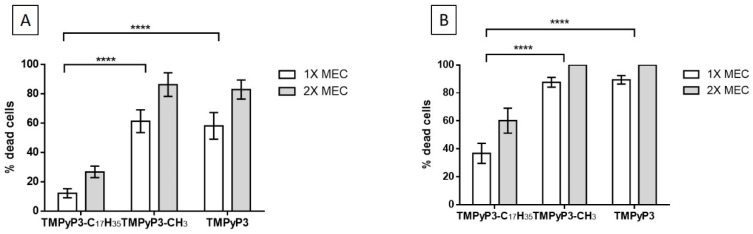
Viability of *Legionella pneumophila* after 10 min (total dose 12 J/cm^2^) (**A**) and 30 min (total dose 36 J/cm^2^) (**B**) exposure to violet light. Data are shown as an average of % dead cells and error bars are representing standard deviation (SD); **** *p* < 0.0001.

Therefore, PDI with porphyrin TMPyP3-C_17_H_35_**,** showed a significantly lower percentage of dead bacteria cells in comparison to hydrophilic porphyrins TMPyP3-CH_3_ and TMPyP3, both after 10 min and 30 min of violet light exposure (Figure 5). No significant difference was observed between hydrophilic porphyrins, tri-cationic TMPyP3-CH_3_ and tetra-cationic TMPyP3. Only amphiphilic porphyrin TMPyP3-C_17_H_35_ showed a strong dose of dependent treatment and increased membrane permeability, with a significantly increased percentage of dead cells after longer (30 min) exposure to violet light. Hydrophilic porphyrins had strong PDI activity already at 1× MEC concentrations, however, their MEC values were 8–16 times higher in comparison to MEC of the amphiphilic porphyrin (Table 3).

All the described data show the percentage of dead cells right after the light exposure. However, 24h after treatment with all three tested porphyrins and exposure to violet light for 10 min, all cells of *L. pneumophila* were dead (data not shown).

In aquatic environments, free-living amoebae (most commonly *Acanthamoeba*, *Hartmannella* and *Naegleria*) serve as host organisms in which *Legionelle* can recover and multiply intracellularly in organelle-studded phagosomes [35,36]. Free-living protozoa can protect intracellular bacteria against disinfectant [37,38]. It has been shown previously that *L. pneumophila* may be rescued after disinfection by the amoebae cells. Garcia et al. have pointed out that amoeba cells are important for the revival of viable-but-nonculturable (VBNC) *L. pneumophila* after disinfection of water with NaOCl [39].

In this work we wanted to test *Acanthamoeba castellanii* in a possible revival of *Legionella* cells after PDI treatment with tested porphyrins. The toxicity of porphyrins using violet light exposure (10 min) on *A. castellanii* was also tested, and it was demonstrated that 50% of the inhibitory concentrations (IC_50_) for all three porphyrins, after exposure to light, were above 10 μM (data not shown).

**Figure 6:**
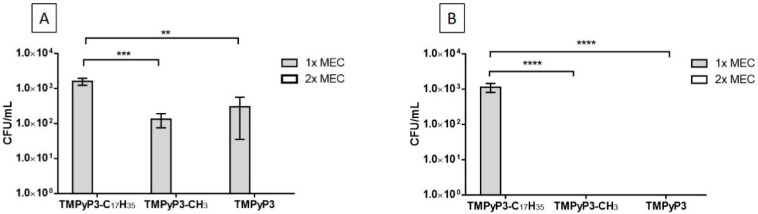
Co-cultivation of *L pneumophila* and *A. castellanii* in the presence of 1× MEC and 2× MEC concentrations of each porphyrin after 10 min (total dose 12 J/cm^2^) (**A**) (control: CFU/mL = 2.45 × 10^8^ ± 2.29) and 30 min (total dose 36 J/cm^2^) (**B**) (control: CFU/mL = 82.14 × 10^8^ ± 2.24) irradiation with violet light. The data are presented as an average of triplicate measurements, and error bars are representing SD; ** *p* = 0.001, *** *p* = 0.002, **** *p* < 0.0001.

*L. pneumophila* treated with PSs was seeded with *A. castellanii* immediately after irradiation for 10 or 30 min, and the control was taken using a similar procedure without a prior treatment of *L. pneumophila* with porphyrins. In comparison to controls (CFU/mL = 2.45 × 10^8^ ± 2.99) with all three porphyrins, a significant decrease was shown in log CFU after 10 min irradiation with violet light. Similar results were obtained after a 30 min irradiation (CFU/mL (control) = 2.14 × 10^8^ ± 2.24), both in control and porphyrin tested samples (Figure 6). All PDI experiments with porphyrins showed a complete inactivation of *L. pneumophila* using 2× MEC concentrations, both after 12 J/cm^2^ of 36 J/cm^2^ of light dose (Figure 6). A significant number of viable bacteria was detected after PDI with TMPyP3-C_17_H_35_ with both doses of light at 1× MEC concentration. This suggests that *L. pneumophila* could recover after such conditions. A stronger PDI activity was demonstrated using hydrophilic porphyrins, TMPyP3 and TMPyP3-CH_3_, and there was a complete eradication of *L. pneumophila* after incubation at 36 J/cm^2^ with both concentrations. After all these PDI treatments *Legionella* did not recover in amoebas after a 24 h incubation (data not shown). Therefore, the recovery of the PDI treated *Legionella* after the treatment depends on the PS type and the time when PDI treated bacteria come into contact with the amoebas.

**Figure 7:**
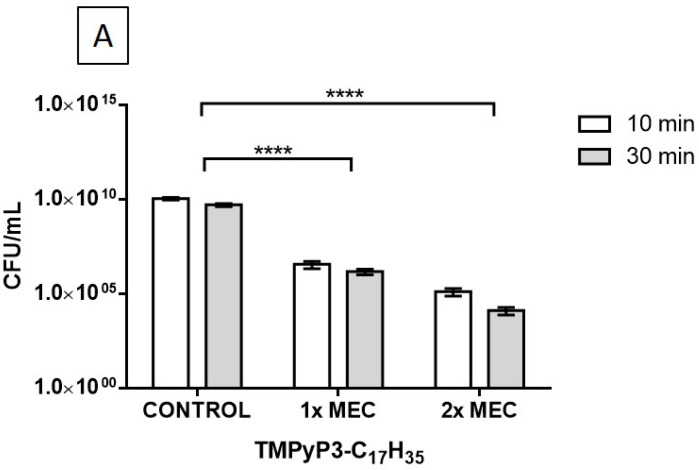

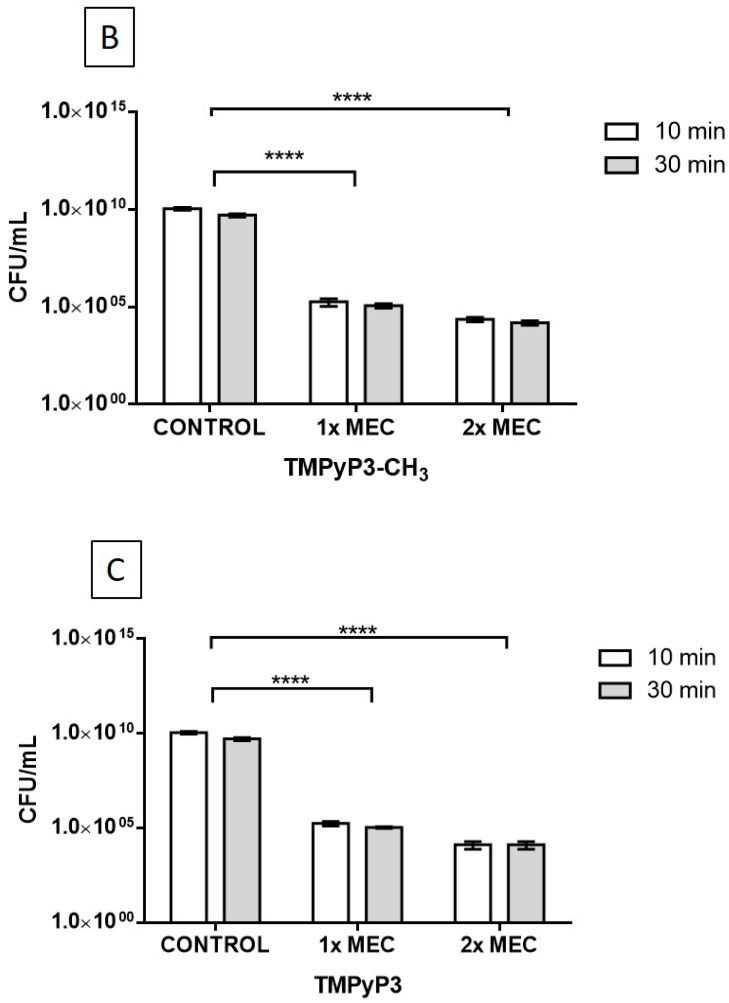
Co-cultivation of *L. pneumophila* and *A. castellanii* in the presence of 1× MEC and 2× MEC concentrations of PSs—porphyrins TMPyP3-C_17_H_35_ (**A**), TMPyP3-CH_3_ (**B**), TMPyP3 (**C**) without exposure to light. The control is the CFU/mLof *L. pneumophila* in the same conditions without PSs. Data are shown as an average of triplicate measurements and error bars represent SD; **** *p* < 0.0001.

On the other side, it can be observed that all tested porphyrins expressed somewhat dark toxicity on *L. pneumophila* that were co-cultivated with *A. castellanii* (Figure 7). In comparison to the controls, all porphyrins showed a significant decrease in the number of *L. pneumophila* after 10 min incubation with 1× MEC concentration of PS (MEC values obtained in PDI experiments, thus upon photoactivation). There was no significant difference in prolonged time of incubation with the same concentration (both for 1× MEC and 2× MEC). By increasing the concentration to 2× MEC, a slightly smaller number of CFU/mL could be observed with all three porphyrins, however it was not a significant difference in comparison to the 1× MEC value (Figure 7).

Interestingly, after treating *L. pneumophila* with porphyrins in the dark (without irradiation), there was no *Legionella*’s multiplication in the observed amoebas, indicating that the tested porphyrins had a certain activity that is independent of light. We are planning to investigate this effect further in our future studies.

## 3. Materials and Methods

### 3.1. General

Chemicals for the synthesis of porphyrins were acquired from Alfa Aesar (Ward Hill, MA, USA), Sigma Aldrich (St. Louis, MO, USA), ACROS organics (Geel, Belgium), VWR Chemicals (Radnor, PA, USA), and thin layer chromatography (TLC) plates and silica-gel from Macherey-Nagel (Düren, Germany). ^1^H NMR spectra were taken at 400 Hz on a NMR spectrophotometer (Bruker Avance III HD) at the Department of Chemistry, University of Zagreb. The absorbance and fluorescence spectra of porphyrins were recorded on Cary 60 UV-Vis and Cary Eclipse respectively, both from Agilent Technologies (Santa Clara, CA, USA). All measurements were carried out using 1 cm quartz cuvette and tap water of the city of Rijeka was used as the solvent. Methylene blue and toluidine blue were acquired from Sigma Aldrich and used as received.

### 3.2. Synthesis of Porphyrins

Porphyrins, 5,10,15,20-tetra(3-pyridinyl)porphyrin (TPyP3), 5-(4-acetamidophenyl)-10,15,20-tetra(3-pyridinyl)porphyrin (TPyP3-CH_3_) and 5-(4-octadecaaminophenyl)-10,15,20-tetra(3-pyridinyl)porphyrin (TPyP3-C_17_H_35_) were synthetized as previously described [19,20]. However, to prepare *N*-methylated derivatives of these porphyrins, we used a modified procedure for methylation according to Ezzedine et al. [21] as follows.

Porphyrin TPyP3-CH_3_ (21 mg, 0.030 mmol) was dissolved in 10 mL dimethylformamide (VWR Chemicals, Radnor, PA, USA) under N_2_. Methyl iodide (Carlo Erba, Barcelona, Spain) (0.2 mL, 0.456 g, 3.213 mmol) was then added and the solution was stirred at RT for 5 h, protected from light. The reaction was checked using TLC on silica plates and CH_3_CN:KNO_3(sat.)_:H_2_O (8:1:1) as the solvent (acetonitrile, CH_3_CN, and potassium nitrite, KNO_3_, were purchased from Sigma Aldrich, St. Louis, MO, USA). When completed, the solvent was removed in vacuo and the reaction mixture dissolved in distilled water. The product was precipitated by adding an aqueous saturated solution of ammonium hexafluorophosphate (NH_4_PF_6_) (Alfa Aesar, Ward Hill, MA, USA) and collected by filtration. The dried precipitate was again dissolved in acetone (VWR Chemicals, Radnor, PA, USA) and tetrabuthylammonium chloride (TBAC) (Sigma Aldrich, St. Louis, MO, USA) was used for another precipitation to obtain chloride salt after filtration and drying. The pure product TMPyP3-CH_3_ was obtained by precipitation from diethyl ether (VWR Chemicals, Radnor, PA, USA) and MeOH (VWR Chemicals, Radnor, PA, USA) (25 mg, 97%), and its ^1^H NMR spectrum (Figure S1) was in full accordance with the literature [19].

The porphyrin TMPyP3-C_17_H_35_ was synthetized using a similar procedure as the one for the porphyrin TMPyP3-CH_3_, starting from the porphyrin TPyP3-C_17_H_35_. The porphyrin TMPyP3-C_17_H_35_ was obtained as brown solid after precipitation from diethyl ether (26 mg, 62%) and the structure was confirmed by ^1^H NMR (Figure S2), which was in complete agreement with the literature [19].

The porphyrin TMPyP3 was synthetized using a similar procedure as the one with the previous two porphyrins, starting from the porphyrin TPyP3.The final product, the porphyrin TMPyP3, was obtained as a brownish solid, after precipitation with Et_2_O (65 mg, 98%) with ^1^H NMR (Figure S3) in full agreement with previous reports [40,41].

### 3.3. Light Source

The light source applied for all experiments was based on a 2D array of high-brightness LED clusters with optical concentrators. The optical design to provide homogeneous irradiation to the sample was produced at the Photonics and Quantum Optics Laboratory and the source was built by the Physical Chemistry Division Workshop at the Ruder Boskovic Institute. The source characterisation and calibration were done using spectroradiometer, (NIST traceable calibrated) providing uniform sample irradiation with 20 mW·cm^−2^ fluence of violet light (*λ*_max_ = 394 nm, Δ*λ*_FWHM_ = 14 nm).

### 3.4. Bacteria Strain and Growth Condition

The photoinactivation studies were conducted with *Legionella pneumophila* serogroup 1, strain ATCC BAA-74 or 130b (clinical isolate). The bacteria were routinely cultured on buffered charcoal yeast extract (BCYE) agar (Oxoid, Altrincham, UK) for 3–5 days at 35 ± 2 °C. They were re-suspended in sterile tap water (STW) and the concentration was adjusted by measuring optical density (OD_600_). A stock solution that contained approximately 10^9^ CFU of *Legionella* per mL was prepared. The bacterial concentrations of approximately 5 × 10^5^ CFU/mL or 1 × 10^8^ CFU/mL were used in the experiments. The number of cultivable bacteria in the experiments was confirmed by enumeration of the bacteria on BCYE-agar.

### 3.5. Tap Water Samples

All photoinactivation studies were performed on tap water from Rijeka (Croatia). For de-chlorination and sterilization, the tap water was sterilized by autoclaving for 15 min at 121 °C and stored at 4 °C until used. The chemical properties of the used tap water were determined using standard methods by the Department of Public Health of Primorsko-goranska county (Table 2).

### 3.6. Determination of the Minimum Effective Concentrations of PSs

The minimum effective concentrations (MEC) of the PSs (MB, TBO, TMPyP3, TMPyP3-CH_3_ and TMPyP3-C_17_H_35_) were determined using a microdilution technique in sterile tap water (STW) as previously described [18]. Briefly, two-fold dilutions of the PSs (50 to 0.043 μM) were mixed with a bacterial suspension (1.0 × 10^5^ CFU/mL) and were incubated for 30 min without light. After that, the bacterial suspension was irradiated for 10 min (12 J/cm^2^). After a 24-h incubation in the dark, samples were plated on BCYE and incubated again for up to 7 days to determine the number of bacteria in the sample. We normally cultivate *Legionella* for 3–5 days, but due to the treatment, the colonies were smaller, thus we extended the incubation for another two days. The MEC value was the lowest concentration of PS that reduces bacterial growth by 99.9%. The dark toxicity of the PSs was also tested in the same way, excluding irradiation of the bacterial suspensions.

### 3.7. PDI Assays in STW

In the experiment, the cell suspensions with ∼10^8^ CFU/mL and the PSs (conc. 2× MEC, 1× MEC and 0.5× MEC) were mixed in STW. After incubation at RT without light and with stirring, the samples were irradiated for different time periods. After each of the photoinactivation treatments, the number of cultivable *Legionella* cells was determined. The results were shown as survival curves of *L. pneumophila* (CFU/mL) depending on the light dose (0–108 J/cm^2^). The results represent the average value of quadruplicate measurements and error bars represent the standard deviation (SD).

### 3.8. Viability of Acanthamoeba castellanii (XTT Assay)

Amoeba cells were seeded in 200 μL of cell suspensions in PYG medium in a concentration of 10^5^ cells/well and incubated for 1 h at 35 ± 2 °C. The PYG medium was then replaced with a PYG medium containing the PSs (TMPyP3, TMPyP3-CH_3_ and TMPyP3-C_17_H_35_) in a concentration range of 0.01–4 μM. After 30 min incubation with porphyrins, the samples were irradiated with violet light for 10 min (the overall light dose was 12 J/cm^2^) and then incubated for 24 h at 35 ± 2 °C, protected from light. The dark control was done simultaneously using the same procedure, but without exposure to light. The next day, plates were treated with XTT and incubated for 2 h. Fluorescence was measured using an ELISA plate reader Fluoromax 3 (Horiba Jobin Yvon, Bensheim, Germany).

### 3.9. Co-Cultivation with Acanthamoeba castellanii

*Legionella pneumophila* was co-cultured with *A. castellanii* in the following manner. Trophozoites of *A. castellanii* were cultivated at 30 °C in PYG medium. The number of *A. castellanii* trophozoites in the experiments was 1 × 10^5^ trophozoites/mL. After the attachment of amoeba to polystyrene, a suspension of 5 × 10^6^ *Legionella* cells/mL was added. The multiplicity of infection (MOI) of bacteria and amoebas was 10:1. After incubation for 2 days at 30 °C, all wells were sonicated for 1 min and the total number of *Legionella* was enumerated by plating on BCYE plates and incubating at 35 ± 2 °C for up to 7 days. During the experiment, the amoeba cell viability was checked using an MTT (3-(4,5-dimethylthiazol-2-yl)-2,5-diphenyltetrazolium bromide) colorimetric assay (Sigma-Aldrich, MO, USA).

### 3.10. Photosensitizer Uptake Assay

As previously described for the uptake assay [18], *Legionella* suspensions with ∼10^8^ CFU/mL in STW were incubated, protected from light, with MEC value of PS, for 30 min. At different time points, to remove free PSs, the bacterial suspension was centrifuged for 10 min (4000 rpm) and the cell pellets were then 2× washed with PBS. For digestion, 2% sodium dodecyl sulfate (SDS) (12 h at RT) and sonication (at 37 °C, 15 min), were used. The concentration of the PSs in the lysed samples was analysed with a Fluoromax 3 (*λ*_exc_ = 422 nm, *λ*_em_ = 651 nm). To obtain the uptake values, the moles (n) of each PSs in the dissolved pellet were divided by the number of CFU in the sample. All data are presented as the average of repeated measurements (5) with standard deviation (SD) in error bars.

### 3.11. Membrane Permeability Assay

Cytoplasmic membrane permeability assays were used (Live/Dead BacLight bacterial viability kit; Invitrogen, Carlsbad, CA, USA), according to the protocol advised by the manufacturer. Briefly, *Legionella* cells grown in ACES-buffered yeast extract (AYE) broth were washed with STW. After 30 min of stirring the bacteria with 1× MEC and 2× MEC values of PSs in the dark, the bacterial cells were treated with PSs at 35 ± 2 °C for 10 and 30 min. Then two nucleic acid stains, propidium iodide (PI) and SYTO-9, were added. After incubation without light for 15 min, microbiological slides were prepared. Digital images were collected using a fluorescence microscope (Olympus BX51, Tokyo, Japan). Four fields, considered to be representative of the entirety of the samples, were selected and the live and dead cells were enumerated manually. Results are presented as an average percentage of dead cells with error bars that represent the standard deviation (SD).

### 3.12. Statistics

The programme GraphPad Prism version 6.0 was used for statistical analyses. All data were expressed as an average of repeated measurements with error bars that represent SD. In all experiments, treatments with different porphyrins were compared using one-way analysis of variance (ANOVA). Tests with a “*p*” value less than 0.05 were regarded as significant.

## 4. Conclusions

We conclude that *Legionella pneumophila* is sensitive to PDI with all the tested cationic porphyrins, with TMPyP3-C_17_H_35_ being the most effective since it causes membrane damage and cell death at the lowest dose. Amphiphilic TMPyP3-C_17_H_35_ has three positive charges for tight binding to a bacterial cell due to electrostatic interaction with negatively charged bacterial LPS, but also has a long alkyl chain as a highly lipophilic part that may penetrate through the membrane. The outer cell structure and membrane lipids have been previously identified as the most important target for PDI, and the combination of both positive charges and lipophilicity in TMPyP3-C_17_H_35_ proved to be the most efficient against *L. pneumophila*.

## Figures and Tables

**Table 1:** General chemical properties of the tap water of the city of Rijeka. All samples were taken from public water supply and analysed.

Parameter	Value
Fuzziness	0.73 NTU
pH value	7.9
Conductivity	0.211 mS/cm at 20 °C
Salinity	0
Hardness	133 mg/L
Anions (HCO_3_^−^, Cl^−^, F^−^, NO_3_^−^, SO_4_^2−^)	148.9 mg/L
Cations (Ca^2+^, Mg^2+^, Na^+^, K^+^)	50.95 mg/L
Hydrocarbons	<1.0 mg/L

**Table 2:** Absorption and fluorescence emission characteristics of porphyrins TMPyP3, TMPyP3-CH_3_ and TMPyP3-C_17_H_35_ (10 µM) in tap water. Molar extinction coefficients (ε) were calculated using the Beer–Lambert law.

Compound	Absorption λ_max_ /nm (ε*10^3^ M^−1^ cm^−1^)					Emission λ_em_/nm (λ_ex_ = 420 nm)
	*Soret*	*Q_y_* (1-0)	*Q_y_* (0-0)	*Q_x_* (1-0)	*Q_x_* (0-0)	
TMPyP3	420 (180.0)	517 (3.9)	546 (10.8)	592 (1.7)	643 (0.2)	608, 657
TMPyP3-CH_3_	420 (204.5)	518 (6.1)	544 (10.2)	596 (1.9)	645 (0.5)	613, 660
TMPyP3-C_17_H_35_	420 (133.2)	518 (3.7)	548 (8.1)	595 (1.4)	644 (0.3)	612, 660

**Table 3:** Minimal effective concentration (MEC) of methylene blue (MB), toluidine blue O (TBO), TMPyP3, TMPyP3-CH_3_ and TMPyP3-C_17_H_35_ on *Legionella pneumophila* in sterile tap water after 10 min using violet light exposure (total light dose 12 J/cm^2^) and in dark conditions (no irradiation).

Compound	MEC (µM)	MEC (µM)-Dark Toxicity
methylene blue (MB)	25.6	>25.6
toluidine blue (TBO)	12.8	>25.6
TMPyP3-C_17_H_35_	0.024	>1.56
TMPyP3-CH_3_	0.39	>12.5
TMPyP3	0.195	>12.5

**Table 4:** Light dose for 50% inhibition (J/cm^2^) of *Legionella* using PSs at 0.5× MEC, 1× MEC and 2× MEC concentrations (MEC values are obtained for all PSs at 12 J/cm^2^ and given in Table 3).

	TMPyP3-C_17_H_35_	TMPyP3-CH_3_	TMPyP3	MB	TBO
0.5× MEC	88.42	26.79	24.55	106.74	104.73
1× MEC	28.96	8.34	9.99	91.62	62.25
2× MEC	24.96	7.36	9.22	72.2	26.72

## Supplementary Materials

Supplementary materials can be found at https://www.mdpi.com/1422-0067/21/15/5367/s1.
