# Supplementary material for: Cationic Porphyrins as Effective Agents in Photodynamic Inactivation of Opportunistic Plumbing Pathogen Legionella pneumophila
Source: Int J Mol Sci. 2020 Jul 28;21(15):5367. doi: 10.3390/ijms21155367 (PMC7432253; doi:10.3390/ijms21155367)
Supplement: Supplementary file 1 [file ijms-21-05367-s001.pdf]

SUPPLEMENTARY INFORMATION

Cationic Porphyrins as Effective Agents in Photodynamic Inactivation of Opportunistic Plumbing Pathogen *Legionella pneumophila*

Andrija Lesar[1], Martina Mušković[2], Gabrijela Begić[3], Martin Lončarić[4], Dijana Tomić Linšak[5], Nela Malatesti[2]* and Ivana Gobin[3]

[1] Bioinstitut d.o.o., R. Steinera 7, 40 000 Čakovec, Croatia

[2] University of Rijeka, Department of Biotechnology, Radmile Matejčić 2, 51000 Rijeka, Croatia

[3] University of Rijeka, Faculty of Medicine, Department of Microbiology and Parasitology, Braće Branchetta 20, 51000 Rijeka, Croatia

[4] Photonics and Quantum Optics Unit, Center of Excellence for Advanced Materials and Sensing Devices, Ruđer Bošković Institute, Bijenička cesta 54, 10002 Zagreb, Croatia

[5] University of Rijeka, Faculty of Medicine, Department for Health Ecology, Braće Branchetta 20, 51000 Rijeka, Croatia

* Correspondence: nela.malatesti@biotech.uniri.hr; Tel. +385-51-584-585; Fax: +385-51-584-599

General

^{1}H NMR spectra were taken at 400 Hz on NMR spectrophotometer (Bruker Avance III HD) at the Department of Chemistry, University of Zagreb. Absorbance spectra of porphyrins were recorded on Cary 60 UV-Vis and Cary Eclipse respectively, both from Agilent Technologies. All measurements were carried out using 1 cm quartz cuvette and tap water of the city of Rijeka, methanol or distilled water was used as solvent.

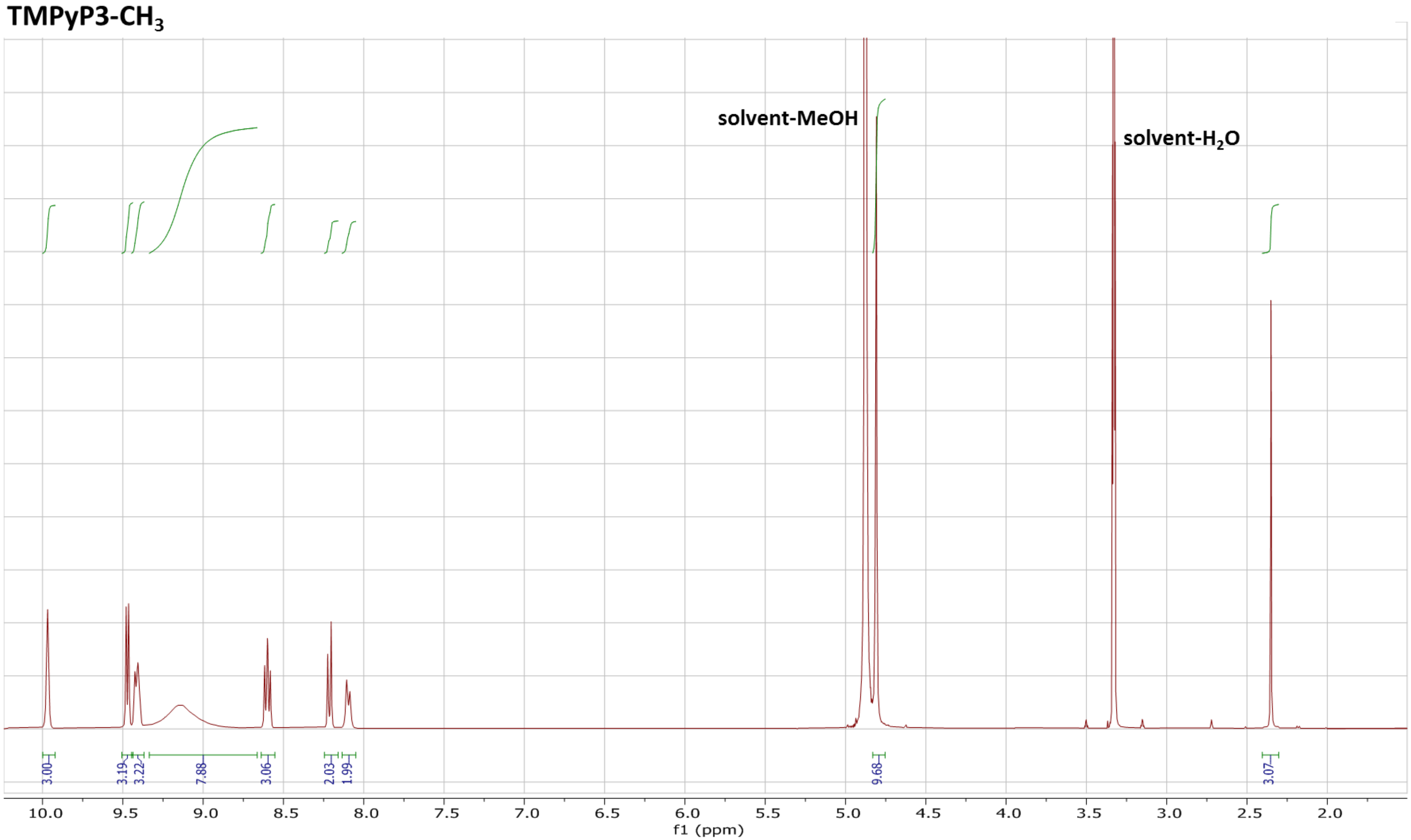

Figure S1. Proton NMR for porphyrin **TMPyP3-CH$_3$** in CD_3OD.

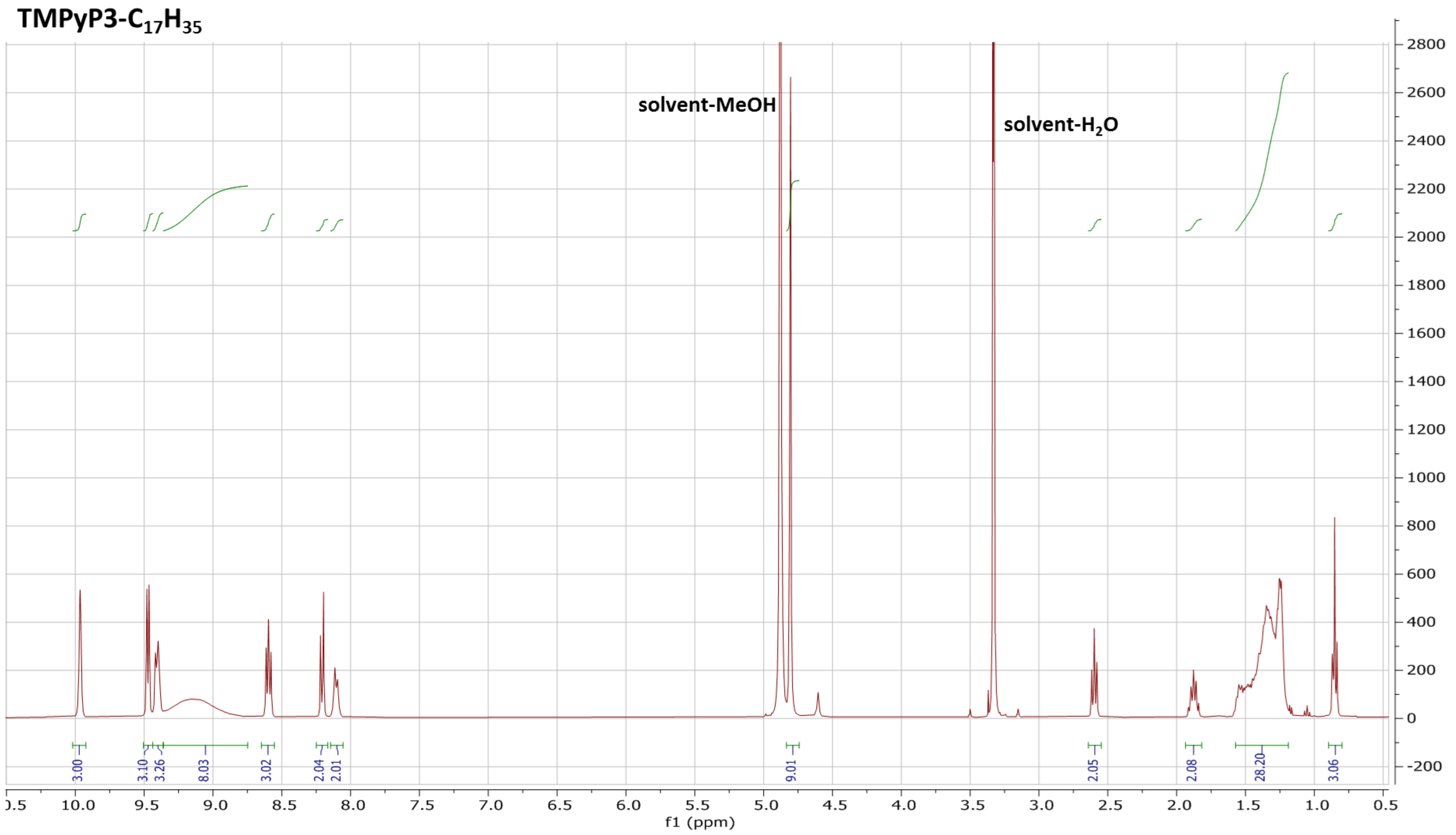

Figure S2. Proton NMR for porphyrin **TMPyP3-$C_{17}H_{35}$** in CD_3OD.

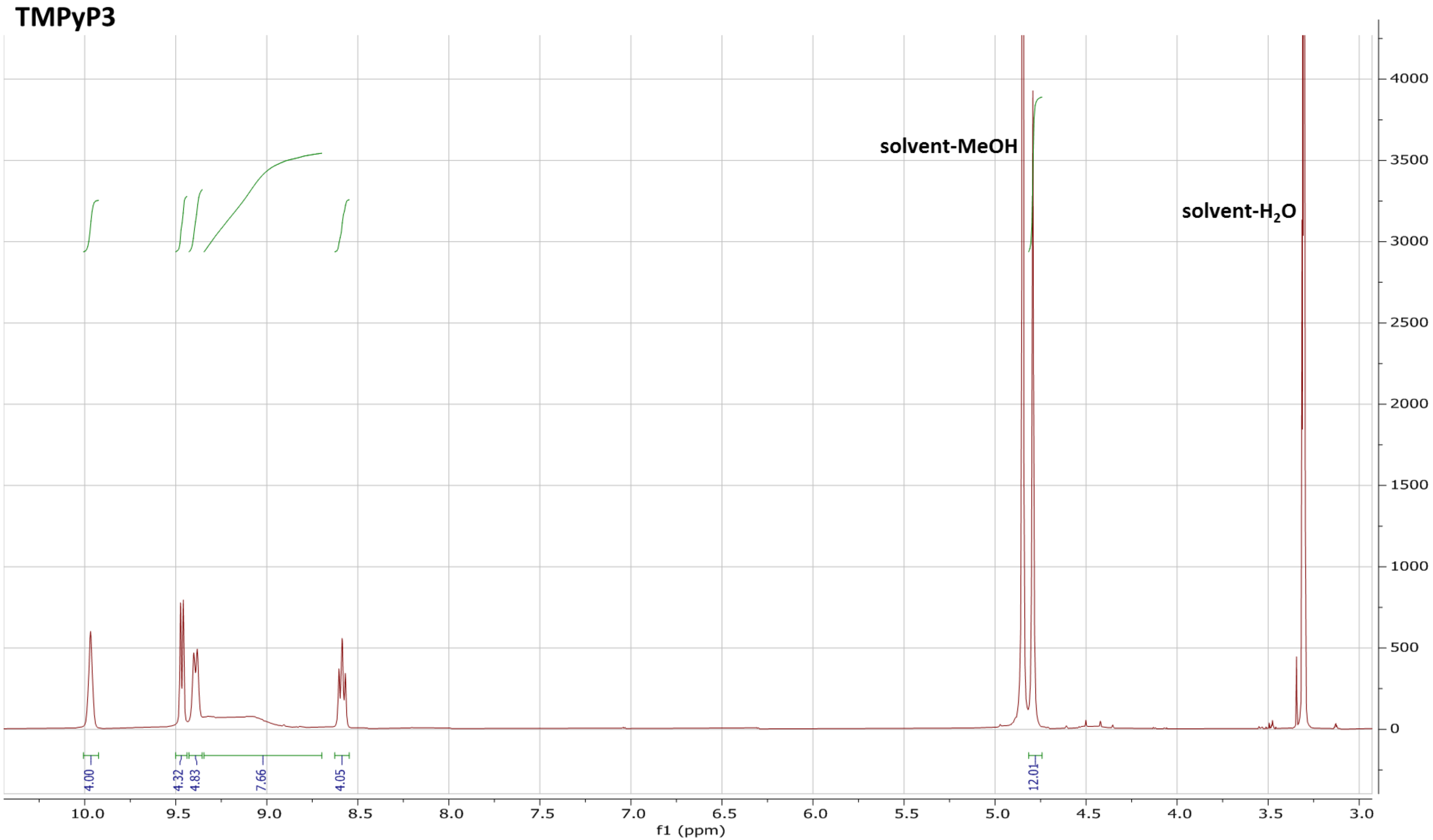

Figure S3. Proton NMR for porphyrin **TMPyP3** in CD_3OD.

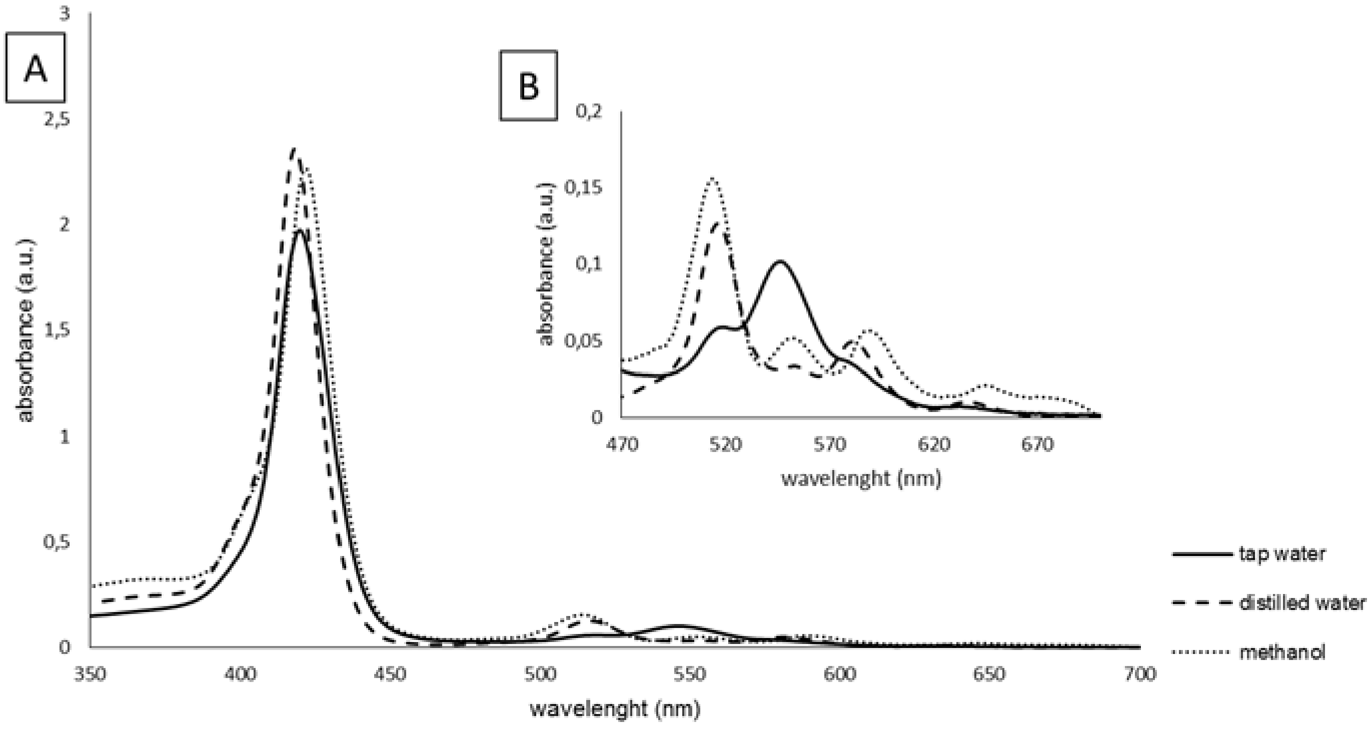

Figure S4. Comparison of **(A)** UV-Vis absorbance spectra and **(B)** Q-bands of same absorption spectra (enlarged), of **TMPyP3-CH_3** (10 μM) in tap water of city of Rijeka, distilled water and methanol.

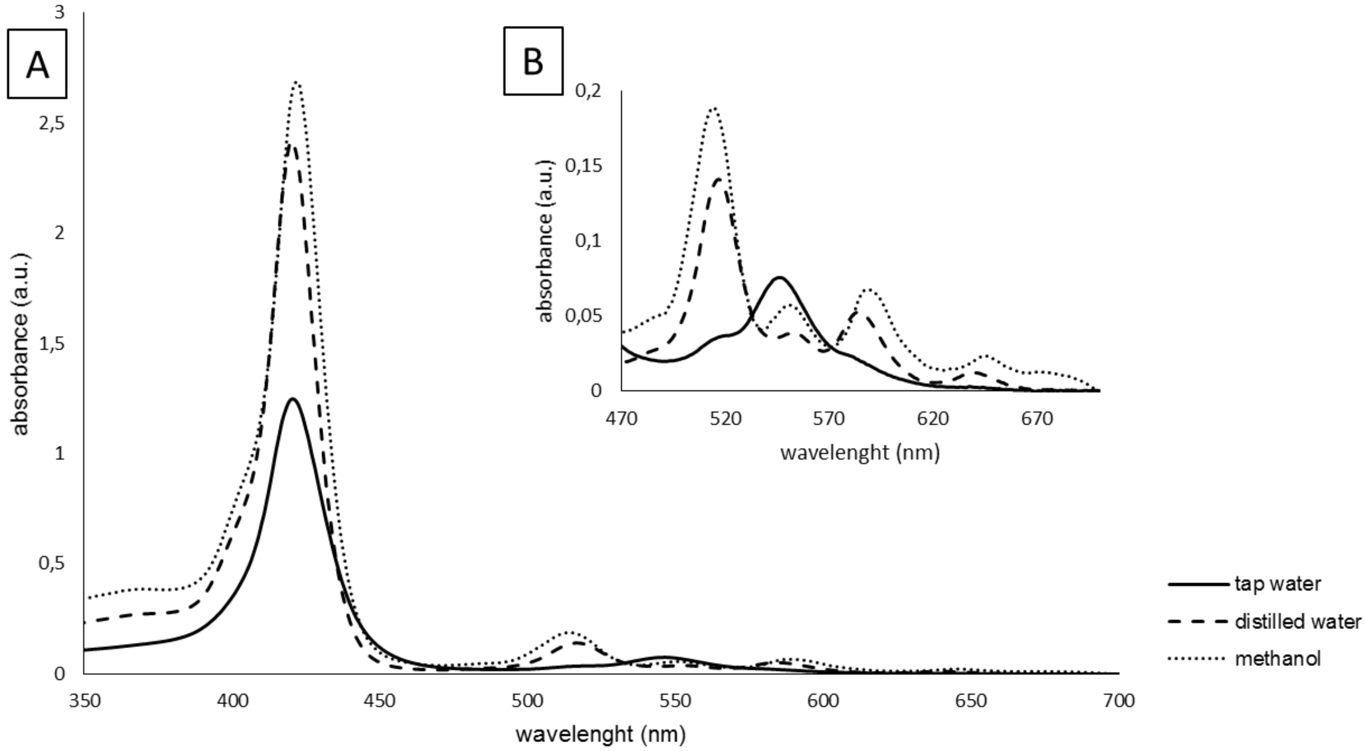

Figure S5. Comparison of **(A)** UV-Vis absorbance spectra and **(B)** Q-bands of same absorption spectra (enlarged), of **TMPyP3-$C_{17}H_{35}$** (10 μM) in tap water of city of Rijeka, distilled water and methanol.

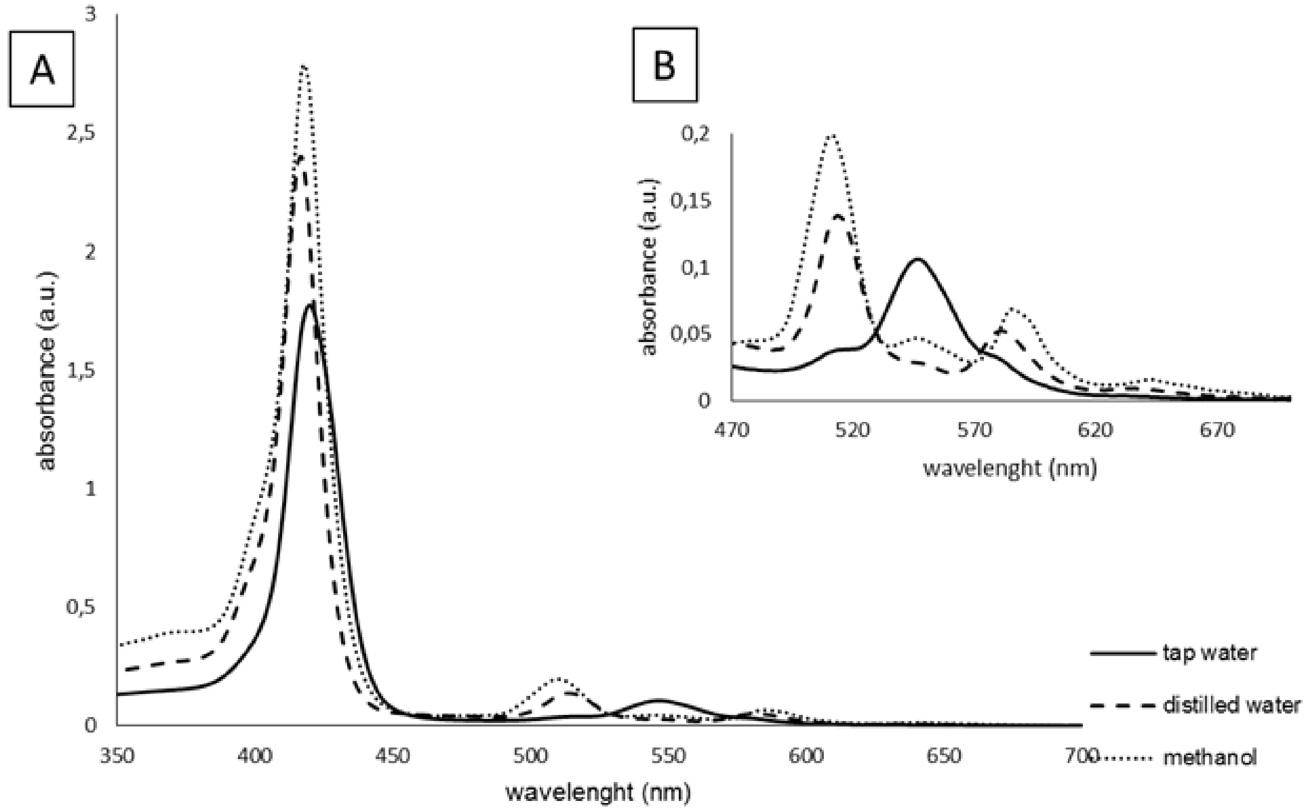

Figure S6. Comparison of **(A)** UV-Vis absorbance spectra and **(B)** Q-bands of same absorption spectra (enlarged), of **TMPyP3** (10 μM) in tap water of city of Rijeka, distilled water and methanol.

Photosensitizer uptake assay

As previously described for the uptake assay [18], *Legionella* suspensions with ~10^8 CFU/mL in STW were incubated, protected from light, with MEC value of PS, for 30 min. At different time points, to remove free PSs, the bacterial suspension was centrifuged for 10 min (4000 rpm) and the cell pellets were then 2X washed with PBS. For digestion, 2% SDS (12 hours at RT) and sonication (at 37 °C, 15 min), were used. The concentration of the PSs in the lysed samples was analysed with a Fluoromax 3 (λ_{exc} = 422 nm, λ_{em} = 651 nm). To obtain the uptake values, the moles (n) of each PSs in the dissolved pellet were divided by the number of CFU in the sample. All data are presented as the average of repeated measurements (5) with standard deviation (SD) in error bars.

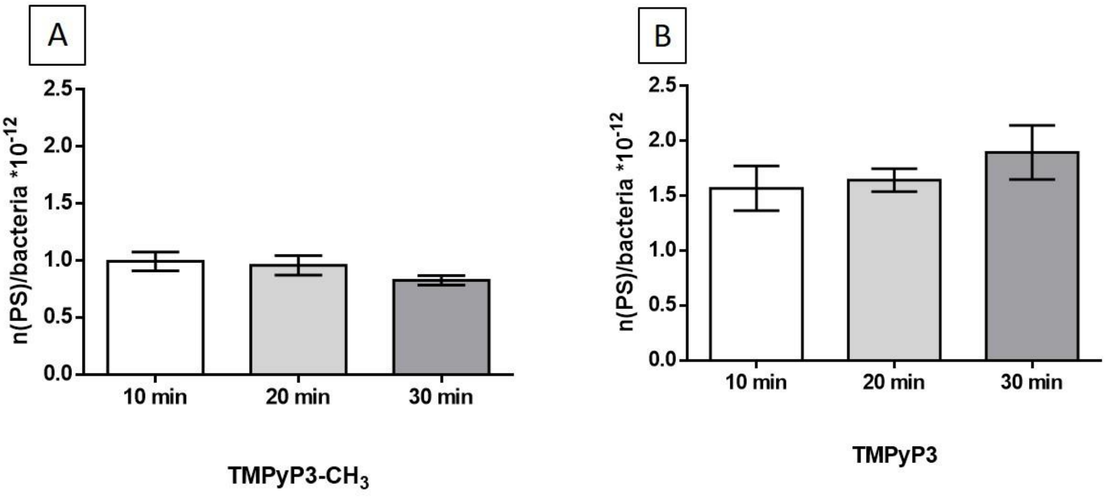

Figure S7. The porphyrin uptake on *Legionella pneumophila* after 10, 20 and 30 min of incubation with concentration of 1x MEC of each porphyrin (**TMPyP3-CH**$_3$ (**A**), **TMPyP3** (**B**)). Data are shown as an average mol (n) of porphyrin per bacteria $\times 10^{-12}$ in 10-, 20- and 30-min time columns. Error bars represent SD.

PDI assays in sterile tap water

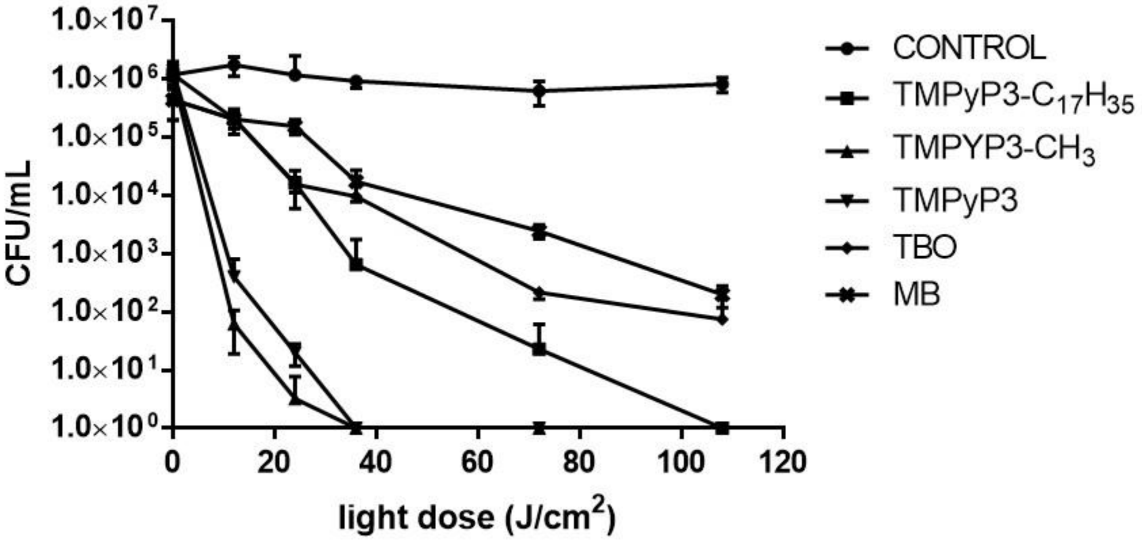

Figure S8. Photodynamic inactivation of *L. pneumophila* (CFU/mL) in the presence of 1x MEC of PSs exposed to different doses of violet light (λ = 394 nm; irradiance 20 mW cm^{-2}). The control is *L. pneumophila* that is exposed to different light doses, without treatment with PSs. Data are given as mean of 3 independent experiments ± SD.

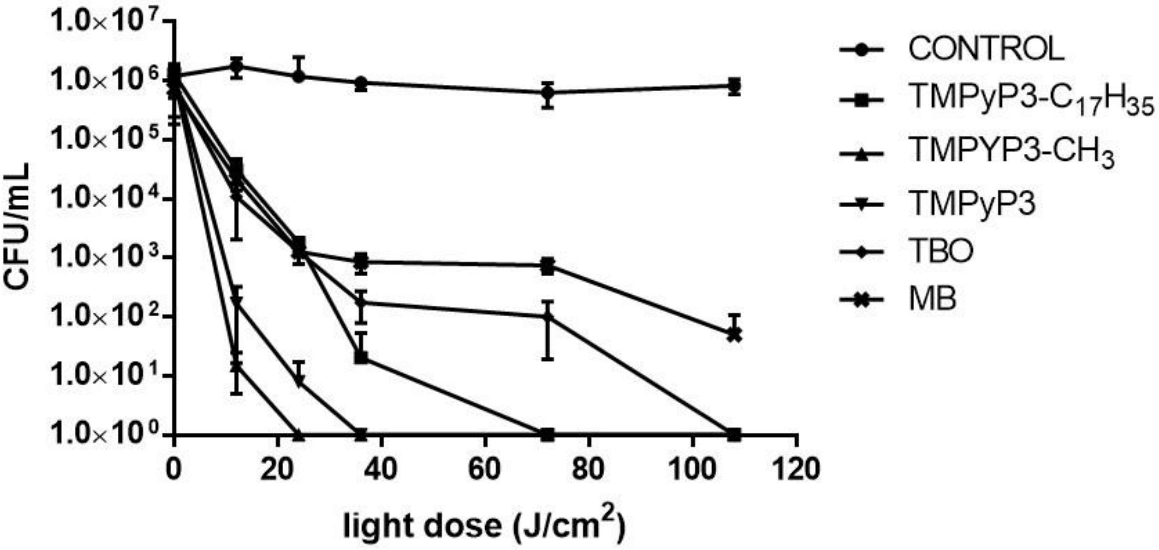

Figure S9. Survival curves of *Legionella pneumophila* (CFU/mL) in the presence of 2x MEC of PSs exposed to different doses of violet light (λ = 394 nm; irradiance 20 mW cm^{-2}). The control is *L. pneumophila* that was exposed to different light doses, without treatment with PSs. Data are given as the mean of 3 independent experiments ± SD.
